# Metabolic Indices of Atherogenicity, Insulin Resistance, and Inflammation (AIP, TyG, and CTI) and the Risk of Renal Function Decline in Early-Stage CKM Syndrome: A Nationwide Prospective Cohort Study

**DOI:** 10.3390/jcm15156055

**Published:** 2026-08-04

**Authors:** Shixin Yan, Haiyang Liu

**Affiliations:** 1 Department of Cardiovascular Surgery, The Second Xiangya Hospital, Central South University, Changsha 410011, China; yansxcsc@163.com; 2 Clinical Center for Gene Diagnosis and Therapy, The Second Xiangya Hospital, Central South University, Changsha 410011, China; 3 Department of Geriatrics, The Second Xiangya Hospital, Central South University, Changsha 410011, China

**Keywords:** cardiovascular–kidney–metabolic syndrome, renal function decline, atherogenic index of plasma, triglyceride-glucose index, C-reactive protein–triglyceride-glucose index, risk stratification

## Abstract

**Background/Objectives:** Cardiovascular–kidney–metabolic (CKM) syndrome, characterized by disturbances in glucose and lipid metabolism as its core pathophysiological feature, is a major driver of target organ damage. While early risk stratification in CKM stages 0–3 is critical to intercept renal decline, the practical utility of accessible metabolic surrogates remains poorly characterized. This study evaluated and compared the prospective capacity of the atherogenic index of plasma (AIP), triglyceride-glucose (TyG) index, and C-reactive protein–triglyceride glucose index (CTI) for predicting renal deterioration in early-stage CKM. **Methods:** Data were extracted from the China Health and Retirement Longitudinal Study (2011–2015). In total, 4064 participants aged ≥ 45 with CKM stages 0–3 (with exclusion of participants with baseline CKM stage 4) were prospectively followed. Renal function decline was defined as an estimated glomerular filtration rate (eGFR) decrease of ≥30% over 4 years, calculated using the CKD-EPI 2012 cystatin C equation. Multivariable logistic regression, restricted cubic splines (RCSs), and receiver operating characteristic (ROC) curves were applied. **Results:** Overall, 122 participants developed the renal outcome. In fully adjusted models, AIP, TyG, and CTI were valid independent risk factors for renal decline, with the lipid-driven AIP exhibiting the strongest association (OR = 5.54, 95% CI: 3.30–9.29; TyG: OR = 1.97, 95% CI: 1.49–2.59; CTI: OR = 1.63, 95% CI: 1.30–2.05; all *p* < 0.001). RCS analysis demonstrated significant non-linear dose–response trajectories, with renal risk escalating sharply past specific exploratory inflection points (AIP = 0.97, TyG = 7.80, CTI = 8.07). ROC curves confirmed robust predictive performance (AUCs > 0.75), with AIP performing optimally (AUC = 0.773). **Conclusions:** AIP, TyG, and CTI are independent longitudinal predictors of renal decline in early-stage CKM, with AIP demonstrating superior discriminatory power. Monitoring these composite indices and maintaining them within observed metabolic ranges may aid in early renal risk stratification and monitoring, though the exploratory statistical inflection points identified require validation in independent cohorts before clinical application.

## 1. Introduction

Cardiovascular–kidney–metabolic (CKM) syndrome, formally defined by the American Heart Association (AHA) in 2023, conceptualizes the complex, bidirectional pathophysiological connections among metabolic disturbances, chronic kidney disease (CKD), and cardiovascular disease (CVD) [1]. Driven by the global epidemic of obesity and related cardiometabolic risk factors, CKM syndrome has a high prevalence among middle-aged and older adults, posing a premier global public health challenge [2,3].

According to the American Heart Association (AHA) staging criteria, Stage 0 CKM is defined by the absence of metabolic risk factors; Stage 1 by dysfunctional adiposity or an excess accumulation of metabolic risk factors; Stage 2 by the emergence of hypertriglyceridemia, hypertension, diabetes, or metabolic syndrome; and Stage 3 by the coexistence of subclinical cardiovascular disease (CVD) or chronic kidney disease (CKD) [4]. The perturbations in glucose and lipid metabolism driven by dysfunctional adipose tissue serve as the central, initiating trigger for CKM-associated multi-organ damage, the pathophysiological essence of which lies in a vicious cycle of chronic energy metabolic imbalance and insulin resistance [1,5,6]. Within this framework, insulin resistance and atherogenic dyslipidemia collectively culminate in irreversible nephron loss through intricately intertwined pathways, including the induction of lipotoxic apoptosis in renal tubular epithelial cells and podocytes, the activation of oxidative stress in glomerular endothelial cells, and the acceleration of renal arteriolosclerosis [7,8,9,10].

Recently, a metabolic–inflammatory review focusing on non-alcoholic fatty liver disease and CKD [11], alongside exploratory investigations into novel biomarkers in metabolic disorders [12], has provided a generalized conceptual background supporting risk-assessment frameworks based on composite metabolic indices. As a critical endpoint of this syndrome, renal function decline not only accelerates the onset of end-stage renal disease (ESRD) but also independently predicts elevated risks of cardiovascular events and all-cause mortality [13,14]. Against this clinical backdrop, a pressing question remains to be answered: can these easily accessible biomarkers of glucose and lipid metabolism prospectively reflect the risk of deteriorating renal function? Validating this hypothesis during CKM Stages 0–3 to identify individuals at high risk for renal decline is of paramount importance for early risk stratification, targeted metabolic monitoring, and ultimately halting the disease progression and the subsequent cascade of organ failure.

Notably, several simple, inexpensive, and easily accessible composite indices have shown substantial potential as surrogate metabolic screening tools in routine clinical practice. Specifically, the atherogenic index of plasma (AIP) sensitively reflects lipid imbalance and the accumulation of small dense LDL (sdLDL) particles associated with atherogenic dyslipidemia [15,16]; the triglyceride-glucose (TyG) index represents a reliable surrogate marker of insulin resistance, closely associated with underlying cardiometabolic dysregulation [17,18,19]; and the recently developed C-reactive protein–triglyceride glucose index (CTI) further integrates systemic inflammation with metabolic pathways [20]. By addressing atherogenicity, insulin resistance, and inflammatory–metabolic statuses from three distinct dimensions, a joint evaluation of these indices promises a more comprehensive risk profile for CKM patients.

However, the precise predictive utility, long-term dose–response trajectories, and potential clinical reference inflection points of these composite metabolic indices within early-stage CKM populations (stages 0–3) remain poorly characterized. Whether their prospective longitudinal association with progressive renal impairment exhibits a strict linear progression or escalates sharply past specific metabolic turning points remains unexamined. This knowledge gap limits their practical application in precise clinical risk stratification, early risk warning, and proactive metabolic management.

To address this gap, this study utilized a nationwide prospective cohort from the China Health and Retirement Longitudinal Study (CHARLS) [21] to evaluate and perform a head-to-head comparison of AIP, TyG, and CTI in predicting renal decline among 4064 participants with CKM stages 0–3. This work not only substantiates the independent predictive value of these accessible metabolic indices but also identifies their distinct non-linear risk inflection points, offering exploratory reference points for early risk stratification before structural kidney damage accelerates.

## 2. Materials and Methods

### 2.1. Study Design and Data Source

This study utilized a longitudinal cohort design based on data extracted from the China Health and Retirement Longitudinal Study (CHARLS), a nationally representative, prospective biennial survey of Chinese adults aged ≥45 years. The CHARLS database contains comprehensive information regarding demographic characteristics, lifestyles, health status, physical examinations, and laboratory biomarkers. For the present study, longitudinal data spanning from the baseline survey in 2011 to the follow-up assessment in 2015 were extracted on 24 December 2025.

**Figure 1:**
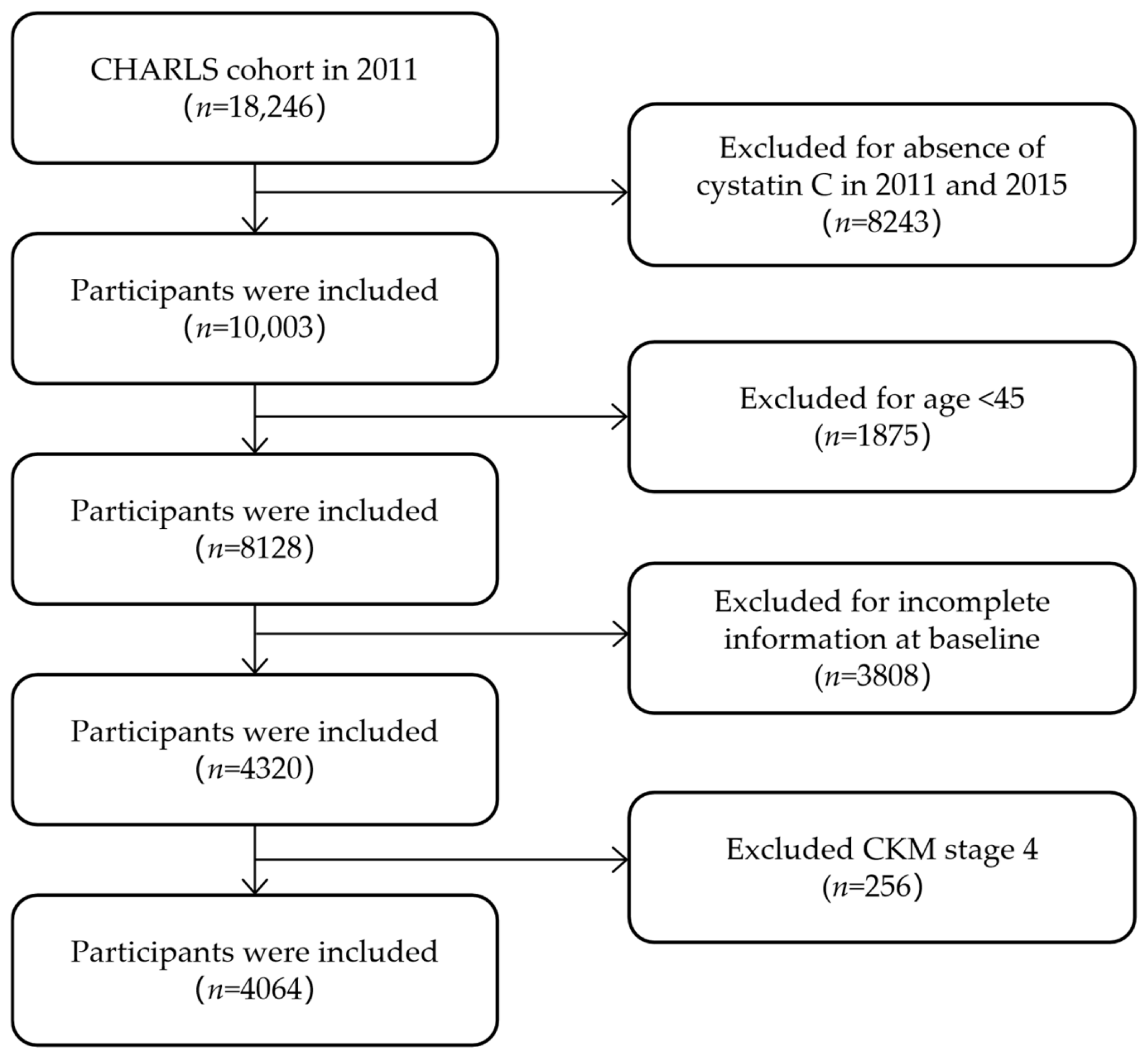
Flowchart of the participant screening process. Abbreviations: CHARLS, China Health and Retirement Longitudinal Study; CKM, cardiovascular-kidney-metabolic.

The baseline survey initially comprised 18,246 individuals. Participants were sequentially excluded if they met any of the following criteria: (1) missing serum cystatin C values for either 2011 or 2015 or (2) missing or invalid essential demographic, physical, or biochemical data. Ultimately, a total of 4064 eligible participants were enrolled in the final analytic cohort. A comprehensive flowchart of the study participant screening process is presented in Figure 1 (Table S1).

### 2.2. Inclusion Criteria and CKM Staging Definitions

Participants were included if they fulfilled all of the following requirements: (1) aged ≥45 years at baseline; (2) participated in both the 2011 and 2015 CHARLS waves with complete follow-up data; and (3) met the clinical criteria for CKM syndrome stages 0 to 3 at baseline, in strict accordance with the 2023 American Heart Association (AHA) scientific statement [1]. To ensure data reproducibility and clinical rigor, we applied a strict operational filtering approach adapted from previous large-scale cohort protocols [22]. The granular inclusion and exclusion criteria, along with the precise physiological thresholds utilized for database screening of each CKM stage, are comprehensively detailed in Table S2.

### 2.3. Data Collection and Variable Measurements

Baseline demographic characteristics (age, sex, marital status, and educational attainment) and lifestyle information (smoking, alcohol consumption, and number of daily meals) were collected using standardized questionnaires administered by interviewers who had undergone uniform training. Specifically, smoking status was determined based on the question in the CHARLS baseline questionnaire: “Have you smoked a total of 100 cigarettes in your lifetime?” Respondents who answered “no” were classified as never smokers, while those who answered “yes” (including current smokers and former smokers who had quit) were classified as ever-smokers. Alcohol consumption status was categorized based on respondents’ self-reported drinking frequency over the past 12 months into three groups: non-drinkers (no alcohol consumption in the past year), less-than-monthly drinkers (drinking but <1 time per month on average), and monthly or more frequent drinkers (≥1 time per month on average). The number of daily meals was derived from the raw variable eating habits in the CHARLS baseline dietary behavior module, which surveyed respondents’ average daily meal frequency. The original questionnaire provided six distinct options (>4 meals/day, 4 meals/day, 3 meals/day, 2 meals/day, 1 meal/day, and <1 meal/day). This study did not merge or recode these categories; instead, the original six-category variable was directly included in the baseline characteristic descriptions and multivariate adjustment models. Physical examinations measured SBP, DBP, height, and weight. BMI was calculated as weight in kilograms divided by the square of height in meters (kg/m^2^).

Venous blood samples were collected after overnight fasting. Laboratory parameters—including total cholesterol, Low-Density Lipoprotein Cholesterol (LDL-C), HDL-C, TG, serum creatinine, blood urea nitrogen (BUN), HbA1c, FPG, uric acid, C-reactive protein (CRP), and serum cystatin C—were determined using automated analyzers via standard laboratory protocols. Estimated glomerular filtration rate (eGFR) was calculated using the CKD-EPI 2012 cystatin C equation [23].

Hypertension was defined as SBP ≥ 130 mmHg, DBP ≥ 80 mmHg, self-reported physician diagnosis, or current use of antihypertensive medications. Diabetes was defined as FPG ≥ 125 mg/dL, HbA1c ≥ 6.5%, self-reported history, or current use of hypoglycemic agents/insulin. Pre-diabetes was defined as an FPG of 100–124 mg/dL or HbA1c of 5.7–6.4% in individuals without diabetes. Hypertriglyceridemia was defined as baseline TG ≥ 135 mg/dL [6,19].

### 2.4. Calculation of Exposure Indices

The three primary composite metabolic exposure indices were calculated at baseline based on well-established mathematical formulas derived from the literature:

Atherogenic Index of Plasma (AIP): Calculated as log10(TG/HDL-C), where both lipid metrics are expressed in molar concentrations (mmol/L) [24].

Triglyceride-Glucose (TyG) Index: Calculated using the formula ln [(TG (mg/dL) × FPG (mg/dL))/2] [17].

C-reactive Protein–Triglyceride Glucose Index (CTI): Calculated as 0.412 × ln (CRP (mg/L)) + ln ((TG (mg/dL) × FPG (mg/dL))/2), integrating systemic inflammation into the metabolic pathway [25].

### 2.5. Outcome Definition

The primary clinical outcome of interest was significant renal function decline over the 4-year follow-up period. Consistent with validated surrogate endpoints for chronic kidney disease progression [26], the outcome event was defined as a decrease in eGFR of more than 30% from the baseline (2011) to the follow-up assessment (2015).

### 2.6. Statistical Analysis

Baseline characteristics were analyzed and stratified by the occurrence of renal function decline. Continuous variables were tested for normality using the Shapiro–Wilk test. Normally distributed continuous data were presented as mean ± standard deviation (SD) and compared using the independent-sample *t*-test. Non-normally distributed continuous variables (including AIP, TyG, CTI, hs-CRP, TG, and FPG) were expressed as median (interquartile range, IQR) and evaluated using the Mann–Whitney U test. Categorical variables were expressed as frequencies and percentages, and compared using the Chi-square (X^2^) test, or Fisher’s exact test where cell counts were limited. To assess the longitudinal associations between the three metabolic indices and renal function decline, three sequential multivariable logistic regression models were constructed: Model 1 (crude model): Unadjusted model evaluating the independent effect of each continuous exposure index (AIP, TyG, or CTI) on the longitudinal renal outcome. Model 2: Model adjusted for key socio-demographic confounders, including baseline age and sex. Model 3 (fully adjusted model): Model further incorporating all remaining potential confounding variables, including marital status, educational attainment, body mass index (BMI), smoking status, alcohol consumption, daily meal frequency, glycated hemoglobin, blood urea nitrogen (BUN), serum uric acid, baseline cardiovascular–kidney–metabolic (CKM) syndrome stage. To further verify the robustness of the results, this study conducted a sensitivity analysis. First, the outcome was defined as a decline in eGFR of ≥20% or ≥40% in separate analyses (Tables S3 and S4), and the fully adjusted model was repeated to examine the consistency of the association under different cutoff values. Second, to address potential bias in the estimates due to rare events, Firth-penalized logistic regression was used to refit the model while maintaining the same covariate adjustment scheme as in the original Model 3. The strength of these prospective associations was quantified using odds ratios (ORs) and 95% confidence intervals (CIs).

To investigate the consistency of the associations between the three metabolic indices and renal function decline across different populations, this study conducted subgroup analyses based on predefined stratification factors, fitting fully adjusted logistic regression models within each stratum. Interaction tests were performed using the likelihood ratio test. *p*-values were calculated by comparing the model fits with and without the product interaction term to determine whether effect modification was present.

This study used a restricted cubic spline (RCS) model to analyze the associations between various exposure factors and outcomes. AIP, CTI, and TyG were included as continuous exposure variables in the RCS model, with four knots set at the 5th, 35th, 65th, and 95th percentiles, respectively. Each model used the median value of each exposure measure as the reference point to estimate the odds ratio (OR) and its 95% confidence interval (CI). The Wald test was used to assess the significance of nonlinear associations. When the RCS model indicated a significant nonlinear association (*p* < 0.05), a single-point piecewise model based on logistic regression was further employed. Using the baseline median of the exposure variable as the initial search value, an iterative algorithm was used to estimate the exploratory statistical inflection points at which the risk trend changed.

Finally, to evaluate the discriminatory capability of the fully adjusted models for each individual index, receiver operating characteristic (ROC) curves were constructed, and the respective areas under the curve (AUCs) with their 95% CIs were computed. At the same time, internal validation was performed using 1000 bootstrap resamples to assess the model’s stability and calculate the area under the curve after optimistic correction.

All statistical calculations were executed using R programming language (version 4.3.0, R Foundation for Statistical Computing, Vienna, Austria). All statistical tests were two-tailed, and a *p*-value < 0.05 was considered statistically significant.

### 2.7. Ethical Statements

This study strictly conformed to the guidelines of the Declaration of Helsinki. The CHARLS project was approved by the Biomedical Ethics Review Committee of Peking University (Ethical Approval Code: IRB00001052-11015; date of approval: 20 January 2011). All participants provided written informed consent prior to data collection.

## 3. Results

### 3.1. Baseline Characteristics

The final analytic cohort comprised 4064 participants, including 122 individuals (3.0%) who experienced significant renal function decline (eGFR decrease ≥ 30%) and 3942 controls (Table 1). Baseline characteristics stratified by the presence of renal function decline are detailed in Table 1. The three primary exposure metrics—AIP, TyG and CTI—demonstrated highly significant differences between the two groups (all *p* < 0.001). Specifically, participants with renal function decline exhibited remarkably higher median baseline levels of AIP (0.64 vs. 0.33), TyG (9.18 vs. 8.64), and CTI (9.40 vs. 8.70) than those in the control group.

Among the adjusted covariates, baseline age, serum uric acid, and hypertension status were profoundly different between the groups (all *p* < 0.001). Notable discrepancies were also observed for marital status (*p* = 0.002), HbA1c (*p* = 0.003), and baseline CKM stage (*p* = 0.002). Compared with the controls, participants in the renal decline group were older, had a higher proportion of widowed individuals (20.5% vs. 11.0%), and demonstrated a greater burden of metabolic complications. Specifically, the prevalence of hypertension (79.5% vs. 60.9%) as well as advanced CKM syndrome (Stage 2: 72.1% vs. 68.6%; Stage 3: 22.1% vs. 14.0%) was significantly enriched in the renal function decline group. The detailed distributions of all adjusted covariates stratified by the presence or absence of renal function decline are presented in Table S5.

To assess selection bias, we compared the baseline characteristics of the included and excluded groups (Table S1). In the inclusion group, age, body mass index, systolic blood pressure, glycated hemoglobin, triglycerides, total cholesterol, and high-density lipoprotein cholesterol levels were all higher than in the exclusion group (*p* < 0.05), while there were no significant differences between the two groups in diastolic blood pressure, serum creatinine, gender, smoking status, or alcohol consumption (*p* > 0.05).

### 3.2. Association of AIP, TyG and CTI with the Risk of Renal Function Decline

Multivariable risk regression analysis revealed that all three primary exposure indices (AIP, TyG and CTI) were significantly associated with an elevated risk of renal function decline across all three sequential models, proving to be independent risk factors (all *p* < 0.05, OR > 1). For the AIP, the risk associations remained robust throughout the adjustments, yielding ORs of 6.52 (95% CI: 4.13–10.28, *p* < 0.001) in Model 1, 7.98 (95% CI: 4.98–12.80, *p* < 0.001) in Model 2, and 5.54 (95% CI: 3.30, 9.29, *p* < 0.001) in the fully adjusted Model 3. Regarding the CTI, the risk of renal decline per unit increase was consistently significant, with ORs of 2.01 (95% CI: 1.67–2.43, *p* < 0.001) in Model 1, 2.03 (95% CI: 1.67–2.46, *p* < 0.001) in Model 2, and 1.63 (95% CI: 1.30–2.05, *p* < 0.001) in Model 3. Similarly, higher TyG index levels were independently coupled with increased odds of the renal outcome, exhibiting ORs of 2.27 (95% CI: 1.82–2.83, *p* < 0.001) in Model 1, 2.47 (95% CI: 1.96–3.10, *p* < 0.001) in Model 2, and 1.97 (95% CI: 1.49–2.59, *p* < 0.001) in Model 3 (Table 2). Furthermore, the results of the sensitivity analysis showed that under two alternative thresholds for defining the outcome—an eGFR decline of ≥20% or ≥40%—AIP, TyG, and CTI all maintained significant and independent associations in the fully adjusted model. This finding further validates the overall consistency of the study’s results across different definitions of the outcome (Tables S3 and S4).

To further control for potential bias in the estimates caused by rare events, we conducted a sensitivity analysis using Firth-penalized logistic regression. While maintaining exactly the same covariate adjustments as in the original Model 3, we refitted the model separately using AIP, TyG, and CTI as the primary exposure variables. The results showed that AIP (OR = 5.53, 95% CI: 3.28–9.30, *p* < 0.001), TyG (OR = 1.96, 95% CI: 1.48–2.58, *p* < 0.001), and CTI (OR = 1.63, 95% CI: 1.29–2.04, *p* < 0.001) all remained significantly positively associated with renal function decline, consistent with the results of the main analysis. We additionally performed a sensitivity analysis by further adjusting the fully adjusted Model 3 for continuous baseline eGFR (Table S6). After fine adjustment for baseline renal function, AIP remained independently associated with renal decline (OR = 2.23, 95% CI: 1.67–2.46, *p* < 0.01), whereas TyG (OR = 1.16, *p* = 0.340) and CTI (OR = 1.18, *p* = 0.172) no longer showed statistically significant independent associations. This indicates that the original independent associations of TyG and CTI were largely dependent on underlying baseline renal function, while AIP retained predictive value independent of precise baseline eGFR.

### 3.3. Restricted Cubic Spline (RCS) Analyses

RCS fitting revealed significant non-linear relationships between the three exposure indices (AIP, TyG, and CTI) and the risk of renal function decline (all *p* for non-linearity < 0.001). The spline curves, combined with their 95% CIs, indicated that as the levels of AIP, TyG, and CTI increased, the ORs initially exhibited a slight downward trend. During this initial phase, the 95% CIs spanned across the 1.0 null line, suggesting that the risk associations were not statistically significant in the low-value range.

**Figure 2:**
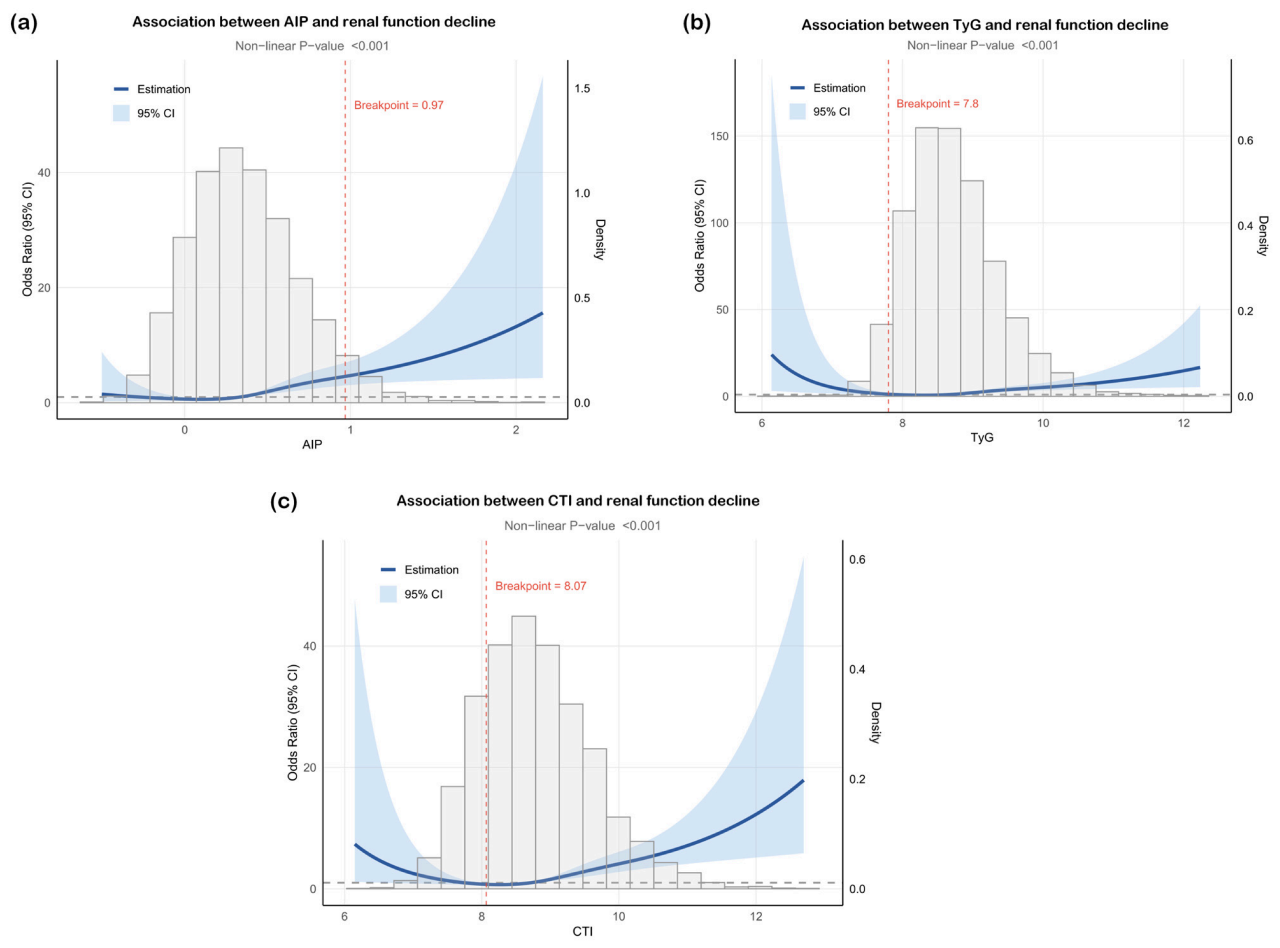
Restricted cubic spline (RCS) curves evaluating the non-linear associations of AIP, TyG, and CTI with the risk of 4-year renal function decline. (**a**) Non-linear trajectory for the atherogenic index of plasma (AIP), showing an exploratory statistical inflection point at 0.97. (**b**) Non-linear trajectory for the triglyceride-glucose (TyG) index, showing an exploratory statistical inflection point at 7.80. (**c**) Non-linear trajectory for the C-reactive protein–triglyceride-glucose (CTI) index, showing an exploratory statistical inflection point at 8.07. Solid blue lines and shaded areas denote the estimated ORs and 95% CIs adjusted for full covariates in Model 3. Overlaid grey histograms represent the density distribution of each composite index within the cohort. The horizontal grey dashed line indicates the reference level (OR=1.0), and the vertical red dashed lines represent the estimated inflection points. Abbreviations: CI, confidence interval; OR, odds ratio.

Subsequently, distinct inflections (turning points) were identified at AIP = 0.97, TyG = 7.80, and CTI = 8.07, where the trajectories inverted from declining to climbing. Beyond these exploratory statistical inflection points, the ORs escalated progressively with higher index levels. Crucially, the corresponding 95% CIs gradually shifted away from the null line (no longer crossing 1.0), indicating a substantial increase in statistical robustness and stability for the positive associations (Figure 2a–c). These findings collectively imply that within this cohort, levels of AIP, TyG, and CTI exceeding their respective inflection points are significantly coupled with an elevated risk of accelerated renal function decline. We further assessed incremental predictive performance, calibration and clinical net benefit of these indices based on a core baseline clinical risk model (Figure S1).

### 3.4. Predictive Performance Evaluation of AIP, CTI, and TyG for Renal Function Decline Risk

**Figure 3:**
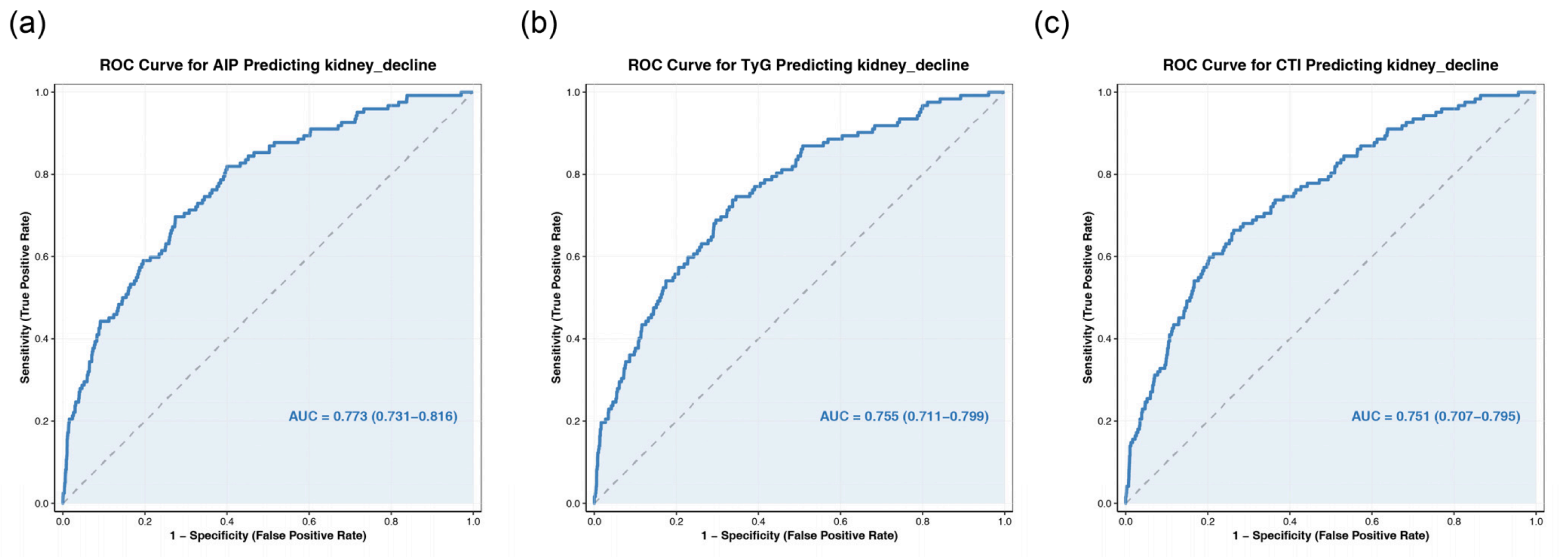
Evaluation of the predictive performance of AIP, TyG, and CTI for the risk of renal function decline. Receiver operating characteristic (ROC) curves are presented for: (**a**) the atherogenic index of plasma (AIP); (**b**) the triglyceride-glucose (TyG) index; and (**c**) the C-reactive protein–triglyceride-glucose (CTI) index. The lipid-driven index (AIP) demonstrated the optimal discriminatory capacity among the three metabolic surrogates. The light blue shaded areas represent the area under the ROC curve (AUC). The diagonal grey dashed line indicates the reference line (AUC = 0.50) representing random chance.

Based on the fully adjusted multivariable model (Model 3), receiver operating characteristic (ROC) curves were generated to evaluate the predictive performance of AIP, TyG, and CTI as primary exposure metrics. The computed areas under the curve (AUCs) were 0.773 (95% CI: 0.731–0.816) for AIP, 0.755 (95% CI: 0.711–0.799) for TyG, and 0.751 (95% CI: 0.707–0.795) for CTI (Figure 3a–c). Following internal validation with 1000 bootstrap resamples, the areas under the curve after optimistic correction were 0.737 for AIP, 0.719 for TyG, and 0.714 for CTI. These statistical values demonstrate that the constructed models possess robust discriminatory and predictive capacity, effectively substantiating the tight risk associations between elevated levels of AIP, TyG, or CTI and the development of significant renal function decline.

## 4. Discussion

Based on a nationally representative prospective cohort of middle-aged and elderly community-dwellers, this study is the first to systematically evaluate and conduct a head-to-head comparison, through a longitudinal follow-up design, of the associations of three composite metabolic indicators—AIP, TyG, and CTI—with the risk of renal function decline across stages 0 to 3 of CKM syndrome, overcoming the limitations of previous cross-sectional or single-indicator studies. Our findings demonstrate that all three indices serve as independent risk factors for renal function decline. Restricted cubic spline (RCS) analysis revealed significant non-linear dose–response curves, characterized by sharp monotonic risk escalation beyond these exploratory statistical inflection points. Furthermore, ROC analysis corroborated their strong discriminatory capacity (all AUCs > 0.75), providing compelling epidemiological evidence for integrating these inexpensive, universally accessible metabolic metrics into CKM-related renal risk stratification.

In the fully adjusted multivariable model, the effect size of AIP (OR = 5.54) substantially outstripped those of TyG (OR = 1.97) and CTI (OR = 1.63), implying that lipid-driven atherogenic pathways exert a dominant role in early metabolic-associated renal injury. This prominent cardiorenal pathological phenomenon aligns with recent longitudinal insights from Chen et al. [27], where lipid-driven markers consistently yielded a more pronounced risk for renal impairment than carbohydrate-driven surrogates. From a pathophysiological perspective, this discrepancy suggests that lipid-driven pathways may play a more dominant role than glucose metabolism pathways in early CKM-related renal injury. Although insulin resistance (reflected by TyG) is widely recognized to impair renal function through pathways such as glucolipotoxicity, oxidative stress, and microinflammation [28], the renal lipotoxicity, glomerular oxidative stress, and renal arteriolosclerosis driven by lipid metabolism dysregulation—represented by AIP—may play a more direct role in early nephron loss [10,29]. Structurally, sdLDL particles easily penetrate the glomerular endothelium and deposit within the mesangium, driving podocyte injury and progressive fibrosis via oxidative stress [30,31]. Meanwhile, in the context of CKM-related metabolic disturbances, HDL-C undergoes dysfunctional alterations, losing its anti-inflammatory and antioxidant capacities [32].

Although the TyG index effectively captures insulin resistance, its downstream pathogenic cascades substantially overlap with lipid-induced lipotoxicity. Consequently, in co-inclusion regression models, the independent effect of TyG as a carbohydrate-load reflection might be partially overshadowed by the dominant lipid-driven impact captured by AIP [17,18,33,34]. Notably, the recent literature has noted that lifestyle-driven metabolic alterations could be closely coupled with downstream metabolic waste retention and serum lipid dysregulation [35]. Interestingly, although CTI incorporates C-reactive protein (CRP) into the TyG framework to simultaneously capture the interactive effects of systemic inflammation and metabolic disorders, its predictive performance did not surpass that of AIP. This suggests that immunometabolic injury in early CKM is primarily rooted in visceral adiposity accumulation—the primary epicenter of chronic low-grade inflammation—rendering the incremental prognostic information provided by systemic CRP relatively limited. Our findings suggest that lipotoxicity, insulin resistance, and microinflammation should not be assessed in isolation; rather, a multidimensional framework for comprehensive cardiometabolic evaluation should be adopted. Future studies may explore additional CKM-specific biomarkers to further elucidate the impact of inflammatory–metabolic interactions on renal function.

Our restricted cubic spline (RCS) analysis revealed that once AIP, TyG, and CTI exceeded their respective exploratory inflection points (0.97, 7.80, and 8.07), the risk of renal function decline exhibited an upward trend. Notably, a recent cross-sectional study based on the U.S. National Health and Nutrition Examination Survey also found a U-shaped association between CTI and albuminuria, with an inflection point of approximately 8.00 [36], which is close to the 8.07 observed in our study, suggesting a degree of consistency of these exploratory statistical inflection points across different populations and renal injury phenotypes. Meanwhile, Shang et al. [37] documented a nonlinear relationship between TyG and renal function deterioration in an inpatient cohort, whereas Chen et al. [27] reported a near-linear dose–response trend using continuous eGFR mathematical slopes in a broader population. This divergence in curve trajectories may reflect a key physiological phenomenon—namely, the distinction between a sustained, low-grade physiological decline and the failure of metabolic compensatory mechanisms. By selectively enrolling early-stage community-dwelling individuals (CKM stages 0–3) rather than those in advanced, institutionalized settings [37], and by adopting a clinically robust endpoint (an absolute eGFR decline of at least 30%) that aligns with consensus hard cardiorenal outcome targets [27], our study observed a turning point in the RCS curves near specific metabolic levels. This phenomenon suggests that the kidneys may maintain considerable metabolic buffering capacity during the early, asymptomatic stages of CKM; however, once metabolic derangement intensifies, such compensatory mechanisms may gradually fail. It should be noted that the reference cut-off for AIP in general cardiovascular risk stratification is approximately 0.11–0.24, whereas the inflection point in our study (0.97) is markedly higher. This is because the inflection point represents a statistical turning point at which the risk trend in the RCS curve shifts from flat to rising, which is fundamentally different from a conventional binary high-risk cut-off, and the two are not directly comparable. In addition, differences in endpoint definitions (renal function decline), renal compensatory reserve, and study population characteristics may also contribute to the upward shift in the inflection points. Therefore, this inflection point should be regarded as an exploratory statistical indicator of elevated renal risk rather than a validated clinical diagnostic threshold or therapeutic target. Moreover, given the limited number of endpoint events in this study (*n* = 122), the estimated values of these inflection points require further validation in independent external cohorts.

These observed exploratory estimates, if validated in future studies, may inform a more proactive approach to risk stratification, complementing current multivariable staging strategies [27,37]. Maintaining AIP, TyG, and CTI within favorable ranges may serve as an adjunctive tool for optimizing metabolic risk assessment, helping to identify high-risk individuals who warrant prioritized attention in the general population. Admittedly, the aforementioned inflection points remain exploratory statistical estimates at present and require validation in independent external cohorts before they can be translated into definitive clinical reference inflection points. Nevertheless, they provide meaningful hypotheses and testable numerical directions for subsequent confirmatory studies, thus holding considerable heuristic value.

The primary strengths of this study include its utilization of a prospective, nationally representative community cohort categorized according to the recent AHA CKM framework, which provides longitudinal insights into the risk profiles of populations in early stages 0 to 3. Nonetheless, certain limitations must be acknowledged. At the data and measurement level, this study only included eGFR data from two time points—the 2011 baseline and the 2015 follow-up—making it impossible to construct repeated-measure trajectories or adopt time-to-event models. Moreover, urinary albumin data were not incorporated, and single eGFR measurements may be susceptible to biological variability, leading to outcome misclassification. In terms of model stability, the inflection points identified by restricted cubic splines were derived from a limited number of events, resulting in insufficient estimation precision at the tails of the exposure distribution; these should be regarded as exploratory statistical inflection points rather than confirmed clinical thresholds. Meanwhile, the effect size of AIP was relatively large and the AUC declined after Bootstrap correction, indicating that its reproducibility requires further evaluation. In addition, given the relatively small number of events, although exploratory subgroup analyses were conducted (Tables S7–S9), the statistical power in highly sparse strata was limited, and the interpretation of specific stratum-specific effect modifications should be approached with caution. With respect to confounding control, this study lacked detailed dietary intake records and key information such as the history of lipid-lowering, glucose-lowering, and antihypertensive medication use, so residual confounding cannot be fully excluded. Regarding population representativeness, the included participants had higher age and metabolic risk profiles than those excluded, and this selection bias may limit the generalizability of the findings to all-age or healthier populations. Future studies should validate the above findings in independent external cohorts and adopt a more intensive follow-up design that incorporates multi-time-point eGFR trajectories and hard endpoints such as urinary albumin, while also collecting standardized food frequency questionnaires and detailed medication history, so as to more comprehensively characterize the dynamic evolution of renal function and enhance the extrapolability of the conclusions.

## 5. Conclusions

In conclusion, this study demonstrates that AIP, TyG, and CTI are valid, independent longitudinal predictors of renal function decline in early-stage CKM syndrome (stages 0–3), with the lipid-driven atherogenic index (AIP) exhibiting the strongest predictive capacity. Our restricted cubic spline analysis confirms distinct non-linear associations centered on exploratory statistical inflection points, which warrant further investigation and external validation before they can be considered for clinical application. From a clinical practice perspective, these simplified and widely accessible surrogate markers hold promise for integration into routine CKM risk stratification, offering statistical reference inflection points to support timely clinical decision-making and metabolic risk monitoring, thereby identifying high-risk individuals before the acceleration of irreversible structural organ damage.

## Figures and Tables

**Table 1:** Baseline characteristics of the study population.

Variables	Categories	Controls (eGFR Decline < 30%) (*n* = 3942)	Renal Decline (eGFR Decline ≥ 30%) (*n* = 122)	*p*-Value
Exposure Indices				
AIP [Median (IQR)]	—	0.33 (0.12, 0.56)	0.64 (0.38, 0.84)	<0.001
TyG index [Median (IQR)]	—	8.64 (8.25, 9.07)	9.18 (8.64, 9.76)	<0.001
CTI [Median (IQR)]	—	8.70 (8.18, 9.28)	9.40 (8.62, 9.98)	<0.001
Demographics & Lifestyles				
Sex [*n* (%)]	Female	2114 (53.6)	66 (54.1)	0.992
	Male	1828 (46.4)	56 (45.9)	
Age (years) [Median (IQR)]	—	59.51 (53.27, 67.02)	65.20 (59.10, 70.87)	<0.001
Educational attainment [*n* (%)]	Junior high school or below	3603 (91.4)	113 (92.6)	0.262
	High school	293 (7.4)	6 (4.9)	
	Technical college or above	46 (1.2)	3 (2.5)	
Marital status [*n* (%)]	Married	3452 (87.6)	93 (76.2)	0.002
	Unmarried	21 (0.5)	1 (0.8)	
	Divorced/separated	35 (0.9)	3 (2.5)	
	Widowed	434 (11.0)	25 (20.5)	
BMI (kg/m^2^) [Median (IQR)]	—	23.13 (20.87, 25.96)	24.32 (21.70, 26.94)	0.007
Smoking status [*n* (%)]	Never smoker	2411 (61.2)	76 (62.3)	0.874
	Ever/current smoker	1531 (38.8)	46 (37.7)	
Alcohol consumption [*n* (%)]	Non-drinker	2663 (67.6)	87 (71.3)	0.661
	<1 time/month	280 (7.1)	7 (5.7)	
	≥1 time/month	999 (25.3)	28 (23.0)	
Daily meal frequency [*n* (%)]	1 time	2 (0.1)	0 (0.0)	0.843
	2 times	514 (13.0)	13 (10.7)	
	3 times	3371 (85.5)	107 (87.7)	
	4 times	42 (1.1)	2 (1.6)	
	>4 times	13 (0.3)	0 (0.0)	
Laboratory Parameters				
HbA1c (%) [Median (IQR)]	—	5.10 (4.90, 5.40)	5.30 (4.93, 5.70)	0.003
BUN (mg/dL) [Mean ± SD]	—	15.82 ± 4.42	15.50 ± 5.57	0.430
Serum creatinine (mg/dL) [Median (IQR)]	—	0.76 (0.66, 0.88)	0.77 (0.67, 0.89)	0.319
Serum uric acid (mg/dL) [Median (IQR)]	—	4.29 (3.56, 5.15)	4.74 (3.92, 5.61)	<0.001
Clinical Comorbidities				
Baseline CKM stage [*n* (%)]	Stage 0	235 (6.0)	2 (1.6)	0.002
	Stage 1	451 (11.4)	5 (4.1)	
	Stage 2	2703 (68.6)	88 (72.1)	
	Stage 3	553 (14.0)	27 (22.1)	
Hypertension status [*n* (%)]	No	1540 (39.1)	25 (20.5)	<0.001
	Yes	2402 (60.9)	97 (79.5)	

Abbreviations: AIP, atherogenic index of plasma; BUN, blood urea nitrogen; BMI, body mass index; CKM, cardiovascular–kidney–metabolic; CTI, C-reactive protein–triglyceride-glucose index; eGFR, estimated glomerular filtration rate; HbA1c, glycated hemoglobin A1c; IQR, interquartile range; TyG, triglyceride-glucose index.

**Table 2:** Association of AIP, CTI, and TyG with renal function decline in three logistic regression models.

Exposure Index	Model 1		Model 2		Model 3	
	OR (95% CI)	*p*-Value	OR (95% CI)	*p*-Value	OR (95% CI)	*p*-Value
AIP	6.52 (4.13, 10.28)	<0.001	7.98 (4.98, 12.80)	<0.001	5.54 (3.30, 9.29)	<0.001
CTI	2.01 (1.67, 2.43)	<0.001	2.03 (1.67, 2.46)	<0.001	1.63 (1.30, 2.05)	<0.001
TyG	2.27 (1.82, 2.83)	<0.001	2.47 (1.96, 3.10)	<0.001	1.97 (1.49, 2.59)	<0.001

Note: Model 1 is unadjusted. Model 2 is adjusted for age and sex. Model 3: Fully adjusted for socio-demographics (marital status and education), lifestyle habits (BMI, smoking, alcohol use, and meal frequency), baseline clinical parameters (HbA1c, BUN, and uric acid), and baseline CKM stage. Abbreviations: AIP, atherogenic index of plasma; CI, confidence interval; CTI, C-reactive protein–triglyceride-glucose index; OR, odds ratio; TyG, triglyceride-glucose index.

## Data Availability

The datasets analyzed during the current study are publicly available from the China Health and Retirement Longitudinal Study (CHARLS) official repository (http://charls.pku.edu.cn/).

## Supplementary Materials

The following supporting information can be downloaded at https://www.mdpi.com/article/10.3390/jcm15156055/s1, Table S1. Comparison of baseline characteristics between included and excluded participants. Table S2. Detailed Operational Criteria and Database Screening Thresholds for Baseline Cardiovascular–Kidney–Metabolic (CKM) Syndrome Stages. Table S3: Sensitivity analysis with the outcome defined as a ≥20% decline in eGFR. Table S4: Sensitivity analysis with the outcome defined as a ≥40% decline in eGFR. Table S5: Baseline covariate distributions by renal function decline event status. Table S6: Sensitivity Analysis—Fully adjusted Model 3 with additional adjustment for continuous baseline eGFR. Table S7: Subgroup Analysis of the Association Between TyG Index and Risk of Renal Function Decline. Table S8: Subgroup Analysis of the Association Between CTI and Risk of Renal Function Decline. Table S9: Subgroup Analysis of the Association Between AIP and Risk of Renal Function Decline. Figure S1. Calibration curves and decision curve analysis (DCA) of the baseline clinical risk model and extended models incorporating AIP, CTI, or TyG (500-bootstrap internal validation).

## Abbreviations

AIP	Atherogenic index of plasma
AUC	Area under the curve
BMI	Body mass index
BUN	Blood urea nitrogen
CHARLS	China Health and Retirement Longitudinal Study
CKD	Chronic kidney disease
CKM	Cardiovascular–kidney–metabolic
CTI	C-reactive protein–triglyceride glucose index
CVD	Cardiovascular disease
eGFR	Estimated glomerular filtration rate
FPG	Fasting plasma glucose
HbA1c	Hemoglobin A1c
RCS	Restricted cubic spline
ROC	Receiver operating characteristic
TyG	Triglyceride-glucose index
WC	Waist circumference
