# Supplementary material for: Metabolic Indices of Atherogenicity, Insulin Resistance, and Inflammation (AIP, TyG, and CTI) and the Risk of Renal Function Decline in Early-Stage CKM Syndrome: A Nationwide Prospective Cohort Study
Source: J Clin Med. 2026 Aug 4;15(15):6055. doi: 10.3390/jcm15156055 (PMC13467047; doi:10.3390/jcm15156055)
Supplement: Supplementary file 1 [file jcm-15-06055-s001.zip › jcm-4379874-supplementary.pdf]

Supplementary Materials

Table S1. Comparison of baseline characteristics between included and excluded participants.

Table S2. Detailed Operational Criteria and Database Screening Thresholds for Baseline Cardiovascular-Kidney-Metabolic (CKM) Syndrome Stages.

Table S3: Sensitivity analysis with the outcome defined as a ⩾20% decline in eGFR.

Table S4: Sensitivity analysis with the outcome defined as a ⩾40% decline in eGFR.

Table S5: Baseline covariate distributions by renal function decline event status.

Table S6: Sensitivity Analysis – Fully adjusted Model 3 with additional adjustment for continuous baseline eGFR.

Table S7: Subgroup Analysis of the Association Between TyG Index and Risk of Renal Function Decline.

Table S8: Subgroup Analysis of the Association Between CTI Index and Risk of Renal Function Decline.

Table S9: Subgroup Analysis of the Association Between AIP Index and Risk of Renal Function Decline.

Figure S1. Calibration curves and decision curve analysis (DCA) of the baseline clinical risk model and extended models incorporating AIP, CTI, or TyG (500-bootstrap internal validation).

Table S1. Comparison of baseline characteristics between included and excluded participants.

Variables	Categories	Overall 18246	Included 4064	Excluded 14182	p
Age, years		58.18 [51.15, 65.63]	59.67 [53.43, 67.32]	57.59 [50.03, 64.87]	<0.001
BMI, kg/m		23.11 [20.80, 25.76]	23.16 [20.89, 26.00]	23.09 [20.77, 25.66]	0.013
Systolic blood pressure, mmHg		127.67 [115.00, 143.33]	129.67 [116.33, 144.33]	127.00 [114.67, 142.67]	<0.001
Diastolic blood pressure, mmHg		75.00 [67.67, 83.67]	75.33 [67.67, 84.00]	75.00 [67.33, 83.67]	0.056
HbA1c, %		5.10 [4.90, 5.40]	5.20 [4.90, 5.40]	5.10 [4.90, 5.40]	<0.001
Triglycerides		106.20 [75.22, 156.65]	107.97 [77.00, 160.18]	105.32 [75.22, 153.99]	0.006
Total cholesterol		189.82 [166.62, 215.34]	192.91 [168.94, 217.37]	187.89 [165.08, 213.79]	<0.001
HDL-C		49.10 [39.82, 59.54]	49.87 [40.98, 60.31]	48.71 [39.43, 59.15]	<0.001
Creatinine		0.76 [0.66, 0.88]	0.76 [0.66, 0.88]	0.76 [0.66, 0.88]	0.441
Sex (%)	Female	8379 (52.4)	2180 (53.6)	6199 (52.0)	0.07
	Male	7606 (47.6)	1884 (46.4)	5722 (48.0)	
Smoking (%)	No	9562 (60.7)	2487 (61.2)	7075 (60.5)	0.438
	Yes	6195 (39.3)	1577 (38.8)	4618 (39.5)	
Drinking (%)	less than once a month	1230 (7.8)	287 (7.1)	943 (8.1)	0.103

Variables	Categories	Overall 18246	Included 4064	Excluded 14182	p
	more than once a month	3899 (24.7)	1027 (25.3)	2872 (24.6)	
	No	10625 (67.4)	2750 (67.7)	7875 (67.4)	

Table S2. Detailed Operational Criteria and Database Screening Thresholds for Baseline Cardiovascular-Kidney-Metabolic (CKM) Syndrome Stages.

CKM Stage	Operational Designation	Component Criteria Required (Any of the following)	Prerequisites / Concurrent Exclusions (All of the following must be met)
**Stage 0**	**No CKM Risk Factors**	**All-Normal Profile:**<br>• BMI < 23.0 kg/m^2<br>• WC < 90 cm (men) or < 80 cm (women)<br>• FPG < 100 mg/dL AND HbA1c < 5.7% (no anti-diabetic meds)<br>• SBP < 130 mmHg AND DBP < 80 mmHg (no anti-hypertensive meds)<br>• TG < 150 mg/dL<br>**• HDL-C ⩾ 50 mg/dL (women) or ⩾ 40 mg/dL (men)**<br>• eGFR ⩾ 60 mL/min/1.73m^2 (no history of CKD)	• Absence of any subclinical or clinical cardiovascular disease (CVD).
**Stage 1**	**Excess Adiposity or Pre-diabetes**	**Early Metabolic Changes:**<br>• Overweight/Obesity (BMI ⩾23.0 kg/m^2)<br>• Abdominal obesity (WC ⩾90/80 cm)<br>• Pre-diabetes (FPG 100–124 mg/dL or HbA1c 5.7%–6.4%)	**Normal Cardio-Renal Profile:**<br>• BP < 130/80 mmHg (no meds)<br>• TG < 150 mg/dL AND **HDL-C normal**<br>• eGFR ⩾ 60 mL/min/1.73m^2 (no CKD)<br>• No subclinical or clinical CVD.
**Stage 2**	**Metabolic Risk Factors or Moderate CKD**	**Established Metabolic/Renal Risk:**<br>• Hypertriglyceridemia (TG⩾ 135mg/dL)<br>• Hypertension (BP⩾130/80 mmHg or on meds)<br>• Diabetes<br>• Metabolic Syndrome (by harmonized criteria)	**No Cardiovascular Target Damage:**<br>• Absence of subclinical or clinical CVD.

		• Moderate CKD (eGFR 30–59 mL/min/1.73m^2 or reported CKD)	
**Stage 3**	**High-Risk Metabolic Disease or Very High-Risk CKD**	**Advanced Subclinical Target Endpoints:**<br><br>• Severe CKD (eGFR < 30 mL/min/1.73m^2)<br><br>• Presence of subclinical CVD (e.g., carotid plaque, ABI < 0.9)	**Free of Overt Clinical Events:**<br><br>• Absence of established clinical CVD (e.g., history of stroke, MI, HF).

Table S3: Sensitivity analysis with the outcome defined as a ≥20% decline in eGFR.

	**Model 1**		**Model 2**		**Model 3**	
**Exposure**	**OR (95% CI)**	**P**	**OR (95% CI)**	**P**	**OR (95% CI)**	**P**
AIP	7.25 (5.16, 10.21)	<0.001	8.16 (5.75, 11.57)	<0.001	6.18 (4.23, 9.04)	<0.001
CTI	2.10 (1.83, 2.41)	<0.001	2.10 (1.83, 2.42)	<0.001	1.82 (1.55, 2.15)	<0.001
TyG	2.47 (2.10, 2.92)	<0.001	2.59 (2.19, 3.06)	<0.001	2.27 (1.86, 2.77)	<0.001

Table S4: Sensitivity analysis with the outcome defined as a ≥40% decline in eGFR.

	**Model 1**		**Model 2**		**Model 3**	
**Exposure**	**OR (95% CI)**	**P**	**OR (95% CI)**	**P**	**OR (95% CI)**	**P**
AIP	8.79 (4.44, 17.37)	<0.001	10.93 (5.39, 22.18)	<0.001	9.96 (4.43, 22.42)	<0.001
CTI	1.99 (1.48, 2.67)	<0.001	1.98 (1.46, 2.68)	<0.001	1.74 (1.21, 2.51)	<0.01
TyG	2.41 (1.72, 3.37)	<0.001	2.62 (1.85, 3.70)	<0.001	2.48 (1.61, 3.82)	<0.001

Table S5: Baseline covariate distributions by renal function decline event status.

**Variable**	**level**	**Total(n)**	**Event(n)**	**Non event (n)**	**event rate**
ckm stage	3	580	27	553	4.66
ckm stage	2	2791	88	2703	3.15
ckm stage	1	456	5	451	1.1
ckm stage	0	237	2	235	0.84
drinking	No	2750	87	2663	3.16
drinking	more than once a month	1027	28	999	2.73
drinking	less than once a month	287	7	280	2.44
education	Above high school	49	3	46	6.12
education	Below high school	3716	113	3603	3.04
education	High school	299	6	293	2.01
gender	Female	2180	66	2114	3.03
gender	Male	1884	56	1828	2.97
hypertension condition	Yes	2499	97	2402	3.88
hypertension condition	No	1565	25	1540	1.6

Variable	level	Total(n)	Event(n)	Non event (n)	event rate
marital status	Previously married	38	3	35	7.89
marital status	Widowed	459	25	434	5.45
marital status	Never married	22	1	21	4.55
marital status	Currently married	3545	93	3452	2.62
meals per day	4	44	2	42	4.55
meals per day	3	3478	107	3371	3.08
meals per day	2	527	13	514	2.47
meals per day	1	2	0	2	0
meals per day	>4	13	0	13	0
smoking	No	2487	76	2411	3.06
smoking	Yes	1577	46	1531	2.92

Table S6: Sensitivity Analysis – Fully adjusted Model 3 with additional adjustment for continuous baseline eGFR.

Exposure Indices	OR (95% CI)	P-value
**AIP**	2.23 (1.67–2.46)	<0.01
**TyG**	1.16 (–)	0.340
**CTI**	1.18 (–)	0.172

Sensitivity logistic regression analysis: Model 3 includes all original covariates (socio-demographics, lifestyle habits, baseline clinical parameters, comorbidities, baseline CKM stage) plus continuous baseline eGFR. Abbreviations: AIP, atherogenic index of plasma; CI, confidence interval; CTI, C-reactive protein-triglyceride-glucose index; OR, odds ratio; TyG, triglyceride-glucose index; eGFR, estimated glomerular filtration rate.

Table S7: Subgroup Analysis of the Association Between TyG Index and Risk of Renal Function Decline.

Subgroup Stratification	Category	Total (N)	Renal Decline Events (n)	Adjusted OR (95% CI)*	P value	P for interaction
**All Participants**	Full Cohort	4,064	122	2.27 (1.82, 2.83)	<0.001	—
**Sex**						0.721
	Female	2,180	66	2.37 (1.75, 3.21)	<0.001	
	Male	1,884	56	2.19 (1.59, 3.01)	<0.001	
**Age (years)**						0.539
	< 60	2,08	31	2.18 (1.49,	<0.001	

		5		3.19)		
	≥ 60	1,979	91	2.53 (1.91, 3.36)	<0.001	
**Education**						0.262
	Below high school	3,716	113	2.38 (1.89, 3.00)	<0.001	
	High school and other	348	9	1.47 (0.66, 3.29)	0.346	
**Marital status**						0.401
	Currently married	3,545	93	2.19 (1.70, 2.81)	<0.001	
	Other	60	4	1.87 (0.49, 7.21)	0.362	
	Widowed	459	25	3.30 (1.87, 5.83)	<0.001	
**Obesity Status (BMI)**						0.885
	< 24.0 kg/m²	2,375	49	2.20 (1.56, 3.09)	<0.001	
	≥ 24.0 kg/m²	1,689	73	2.27 (1.67, 3.09)	<0.001	
**Smoking**						0.522
	No	2,487	76	2.16 (1.63, 2.85)	<0.001	
	Yes	1,577	46	2.51 (1.74, 3.60)	<0.001	
**Drinking**						0.147
	Less than once a month	287	7	1.03 (0.37, 2.86)	0.952	
	More than once a month	1,027	28	2.06 (1.34, 3.16)	0.001	
	No	2,750	87	2.57 (1.96, 3.38)	<0.001	

**Meals per day**						0.003
	3 meals/day	3,478	107	2.58 (2.04, 3.25)	<0.001	
	Other	586	15	0.72 (0.32, 1.61)	0.420	
**Glycemic Status (HbA1c)**						0.244
	< 6.5%	3,849	110	2.30 (1.76, 3.00)	<0.001	
	≥ 6.5%	215	12	1.59 (0.91, 2.78)	0.102	
**Baseline CKM Stage**						0.406
	0	237	2	0.41 (0.01, 19.41)	0.648	
	1	456	5	1.56 (0.14, 17.15)	0.715	
	2	2,791	88	1.93 (1.47, 2.53)	<0.001	
	3	580	27	2.92 (1.80, 4.75)	<0.001	

Table S8: Subgroup Analysis of the Association Between CTI Index and Risk of Renal Function Decline.

Subgroup Stratification	Category	Total (N)	Renal Decline Events (n)	Adjusted OR (95% CI)*	P value	P for interaction
**All Participants**	Full Cohort	4,064	122	2.01 (1.67, 2.43)	<0.001	—
**Sex**						0.941
	Female	2,180	66	2.01 (1.55, 2.59)	<0.001	
	Male	1,884	56	2.03 (1.54, 2.69)	<0.001	
**Age (years)**						0.921
	< 60	2,08	31	2.00 (1.43,	<0.001	

		5		2.79)		
	≥ 60	1,97 9	91	2.04 (1.61, 2.58)	<0.001	
**Education**						0.893
	Below high school	3,71 6	113	2.02 (1.66, 2.47)	<0.001	
	High school and other	348	9	1.93 (1.01, 3.71)	0.048	
**Marital status**						0.782
	Currently married	3,54 5	93	1.98 (1.60, 2.46)	<0.001	
	Other	60	4	1.63 (0.48, 5.58)	0.432	
	Widowed	459	25	2.30 (1.49, 3.56)	<0.001	
**Obesity Status (BMI)**						0.833
	< 24.0 kg/m²	2,37 5	49	1.95 (1.47, 2.59)	<0.001	
	≥ 24.0 kg/m²	1,68 9	73	2.04 (1.55, 2.67)	<0.001	
**Smoking**						0.246
	No	2,48 7	76	1.85 (1.46, 2.35)	<0.001	
	Yes	1,57 7	46	2.34 (1.71, 3.21)	<0.001	
**Drinking**						0.142
	Less than once a month	287	7	0.93 (0.39, 2.24)	0.869	
	More than once a month	1,02 7	28	2.01 (1.38, 2.94)	<0.001	
	No	2,75 0	87	2.16 (1.72, 2.71)	<0.001	
**Meals per day**						0.025

	3 meals/day	3,478	107	2.18 (1.79, 2.66)	<0.001	
	Other	586	15	1.05 (0.57, 1.92)	0.881	
**Glycemic Status (HbA1c)**						0.407
	< 6.5%	3,849	110	1.99 (1.59, 2.48)	<0.001	
	≥ 6.5%	215	12	1.57 (0.95, 2.61)	0.077	
**Baseline CKM Stage**						0.214
	0	237	2	0.88 (0.08, 10.15)	0.917	
	1	456	5	0.54 (0.10, 2.94)	0.473	
	2	2,791	88	1.80 (1.43, 2.26)	<0.001	
	3	580	27	2.47 (1.63, 3.75)	<0.001	

*Renal function decline was defined as a ⩾30% decrease in the estimated glomerular filtration rate (eGFR) over the 4-year follow-up period. Models are mutually adjusted for age, sex, education, marital status, BMI, smoking status, alcohol consumption, daily meal frequency, HbA1c, BUN, serum creatinine, serum uric acid, baseline CKM stage, and hypertension condition, except for the variable used for stratification itself. Abbreviations: AIP, atherogenic index of plasma; CI, confidence interval; CTI, C-reactive protein-triglyceride glucose index; eGFR, estimated glomerular filtration rate; OR, odds ratio; TyG, triglyceride-glucose index.

Table S9: Subgroup Analysis of the Association Between AIP Index and Risk of Renal Function Decline.

Subgroup Stratification	Category	Total (N)	Renal Decline Events (n)	Adjusted OR (95% CI)*	P value	P for interaction
**All Participants**	Full Cohort	4,064	122	6.52 (4.13, 10.28)	<0.001	—
**Sex**						0.419
	Female	2,180	66	7.84 (4.16, 14.79)	<0.001	

	Male	1,884	56	5.38 (2.79, 10.38)	<0.001	
**Age (years)**						0.428
	< 60	2,085	31	5.82 (2.65, 12.78)	<0.001	
	≥ 60	1,979	91	8.65 (4.82, 15.55)	<0.001	
**Education**						0.273
	Below high school	3,716	113	7.15 (4.42, 11.55)	<0.001	
	High school and other	348	9	2.80 (0.56, 13.94)	0.208	
**Marital status**						0.427
	Currently married	3,545	93	6.09 (3.63, 10.22)	<0.001	
	Other	60	4	4.18 (0.33, 52.21)	0.267	
	Widowed	459	25	13.22 (4.31, 40.60)	<0.001	
**Obesity Status (BMI)**						0.838
	< 24.0 kg/m^2	2,375	49	6.16 (3.12, 12.17)	<0.001	
	≥ 24.0 kg/m^2	1,689	73	6.80 (3.51, 13.18)	<0.001	
**Smoking**						0.784
	No	2,487	76	6.23 (3.51, 11.06)	<0.001	
	Yes	1,577	46	7.11 (3.34, 15.13)	<0.001	
**Drinking**						0.060
	Less than once a month	287	7	0.73 (0.08, 6.49)	0.781	
	More than once a	1,027	28	5.81 (2.47, 13.66)	<0.001	

	month					
	No	2,750	87	8.49 (4.80, 15.03)	<0.001	
**Meals per day**						0.102
	3 meals/day	3,478	107	7.71 (4.70, 12.64)	<0.001	
	Other	586	15	2.40 (0.65, 8.88)	0.189	
**Glycemic Status (HbA1c)**						0.067
	< 6.5%	3,849	110	7.08 (4.27, 11.75)	<0.001	
	≥ 6.5%	215	12	2.16 (0.67, 6.93)	0.197	
**Baseline CKM Stage**						0.454
	0	237	2	6.31 (0.00, 14834.65)	0.642	
	1	456	5	3.77 (0.03, 486.30)	0.592	
	2	2,791	88	4.74 (2.74, 8.21)	<0.001	
	3	580	27	12.94 (4.28, 39.14)	<0.001	

Figure S1. Calibration curves and decision curve analysis (DCA) of the baseline clinical risk model and extended models incorporating AIP, CTI, or TyG (500-bootstrap internal validation).

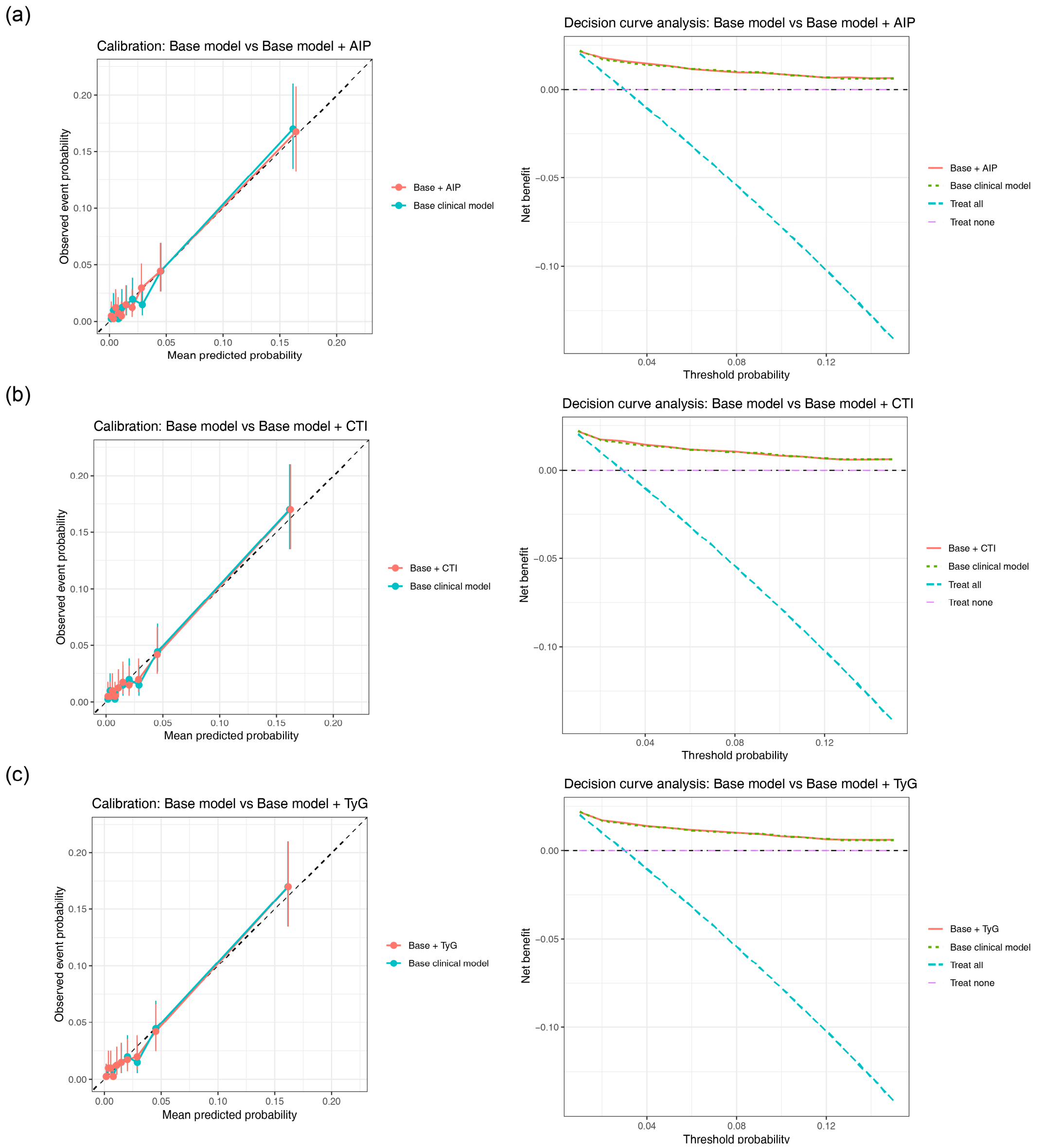

(a) Base clinical model vs Base model + AIP (b) Base clinical model vs Base model + CTI (c) Base clinical model vs Base model + TyG The baseline clinical model includes age, gender, continuous baseline eGFR, CKM stage, hypertension, diabetes, uric acid, HbA1c, and conventional lipid/glucose markers. Abbreviations: AIP, atherogenic index of plasma; CTI, C-reactive protein-triglyceride-glucose index; TyG, triglyceride-glucose index; DCA, decision curve analysis; eGFR, estimated glomerular filtration rate.
